# James Joyce. Chronisch rezidivierende Iritis infolge einer postvenerischen reaktiven Arthritis

**DOI:** 10.1007/s00393-024-01614-8

**Published:** 2025-02-12

**Authors:** H. Zeidler

**Affiliations:** https://ror.org/00f2yqf98 grid.10423.34 0000 0000 9529 9877 Ehm. Klinik für Rheumatologie, Medizinische Hochschule Hannover, Carl-Neuberg Str. 1, 30625 Hannover, Deutschland

**Keywords:** Pathobiographie, Sehverlust, Gelenkerkrankung, Differentialdiagnose, Pathobiography, Loss of vision, Joint disease, Differential diagnosis

## Abstract

Der irische Autor James Joyce (1882–1941) erkrankte an chronisch rezidivierender Iritis mit vielfältigen Komplikationen, die zu einer fast völligen Blindheit führten. Als Auslöser diskutiert wurden v. a. Syphilis und eine rheumatische Erkrankung im Sinne eines Reiter-Syndroms. Das zeitliche Zusammentreffen von venerischer Infektion, Arthritis und charakteristischen, ärztlich dokumentierten Merkmalen der Iritis einschließlich der typischen Komplikationen sind zusammen mit dem chronisch rezidivierenden Verlauf der Augenerkrankung überzeugende Argumente für eine postvenerische reaktive Arthritis. Für den ungünstigen Verlauf der Iritis sind möglicherweise eine familiäre Disposition und Rauchen mit verantwortlich. Es fehlen sichere Hinweise auf eine Syphilis, und v. a. die mehrfachen Rezidive der Iritis sind nicht vereinbar mit einer luetischen Infektion. Kunsthistoriker haben auch nach den medizinischen Publikationen, in denen die rheumatische Genese überzeugend diskutiert wurde, und in deren Kenntnis an der Diagnose der Syphilis festgehalten. Deshalb ist zu wünschen, dass zukünftig die kunsthistorische Literatur die Krankheit von Joyce entsprechend der medizinischen Erkenntnisse wiedergibt.

## Abstract (en)

The Irish author James Joyce (1882–1941) suffered from chronic recurrent iritis with a variety of complications that led to almost complete blindness. Syphilis and a rheumatic disease, such as Reiter’s syndrome were discussed as the main causes. The coincidence of a venereal infection, arthritis and the characteristic medically documented features of iritis, including typical complications, together with the chronic recurrent course of the eye disease, are convincing arguments for postvenereal reactive arthritis. A familial predisposition and smoking could have been responsible for the unfavorable course of the iritis. There is no reliable evidence of syphilis and, above all, the multiple recurrences of the iritis are not compatible with a syphilitic infection. Even after the medical publications in which the rheumatic genesis was convincingly discussed and in the knowledge of these, art historians have held on to the diagnosis of syphilis. It is therefore to be hoped that in the future the art historical literature will accurately reflect Joyce’s illness in accordance with the medical knowledge.

Der irische Autor James Joyce (1882–1941) gilt wegen seines experimentellen Sprachgebrauches und der Erforschung neuer literarischer Methoden als einer der einflussreichsten und bedeutendsten Schriftsteller des 20. Jahrhunderts. Er studierte moderne Sprachen, schloss das Studium mit einem Bachelor of Art ab und wollte Schriftsteller werden. Um seinen Lebensunterhalt beim Schreiben zu bestreiten, nahm er in Dublin und Paris ein Medizinstudium auf, das er nach kurzer Zeit wieder aufgab [18]. Seine Werke, insbesondere „The Dead“, „A Portrait of the Artist as a Young Man“, „Ulysses“ und „Finnegans Wake“, sind stark autobiografisch und beinhalten sehr genaue Beschreibungen von Krankheiten [8]. Joyce selbst erkrankte an chronisch rezidivierender Iritis mit vielfältigen Komplikationen, deren Ursache zu seinen Lebzeiten letztlich unklar blieb. Als Auslöser vermutet wurden Syphilis, rheumatisches Fieber, Zahnkaries und Malaria [12, 24].

Der irische Arzt und Medizinhistoriker John Lyons diskutierte erstmals die Diagnose des Reiter-Syndroms bzw. einer ankylosierenden Spondylitis aufgrund der Auswertung der Briefe von Joyce und anderer Dokumente [12]. Die folgende pathobiografische und medizinhistorische Betrachtung bewertet vor dem Hintergrund der heutigen Klassifikation der Spondyloarthritiden (SpA), ob die Erkrankung von Joyce als reaktive Arthritis (ReA) interpretiert werden kann.

## Augenerkrankung

Joyce erkrankte erstmals 1907 an einer Iritis links, die zu zahlreichen Rezidiven führte (Tab. 1). Als Folge kam es zu Synechien, Glaukom und Katarakt, die operativ behandelt wurden, teilweise mit Komplikationen (Glaskörperverlust und Blutungen). Auch Joyces rechtes Auge wurde von einer Iritis betroffen und führte im Verlauf zu einer totalen, verkalkten Katarakt mit sekundärem Glaukom, teilweiser Atrophie der Netzhaut und des Sehnervs. Er wurde mit kalten Umschlägen, Dionin, miotischen Augentropfen und mit Arsen- und Phosphorinjektionen behandelt. Als die modernste Behandlung der Zeit erhielt er kleine Blutegel zur Extraktion von Blut aus der vorderen Augenkammer [1]. Joyce unterzog sich zahlreichen Augenoperationen (Iridektomie, Sphinkterektomie, Kataraktextraktion und Kapsulotomie) mit dem letztlich vergeblichen Ziel, sein Sehvermögen zu bewahren [1]. Trotz der in seiner Zeit verfügbaren konservativen und operativen Behandlungen wurde seine Sehfähigkeit immer schlechter. Er war schließlich auf dem rechten Auge blind, und das linke Auge hatte nur noch 10 % Sehkraft [8]. Joyce konnte dadurch kein einziges gedrucktes Wort mehr lesen, es gelang ihm nur, mit einem schwarzen Bleistift in großen schwarzen Buchstaben zu schreiben und mit einer Lupe geschriebene Wörter zu entziffern [1].

**Tab. 1:** Die Augenmanifestationen und Therapie. (Zusammengestellt aus [11, 22, 24, 26])

Jahr	Manifestation	Therapie
1907	Iritis links	Silbernitrat
1908	Rezidiv Iritis links	–
1909	Rezidiv Iritis links	–
1917	Rezidiv Iritis links mit Synechien und Glaukom	Iridektomie. Postoperative Blutung
1918	Iritis beidseits mit Glaukom	–
1919	Rezidiv Iritis links	–
1920	Rezidiv Iritis links	Miotische Augentropfen. Kalte Kompressen
1921	Rezidive Iritis links	–
1922	Schweres Rezidiv Iritis linkes Auge	Blutegel wurden verwendet, um eine konjunktivale Stauung zu reduzieren. Dionin-(Ethylmorphin‑)Augentropfen
1923	Rezidiv Iritis links, Iritis rechts	Sphinkterektomie
1924	Rasch progrediente Katarakt links	Operation der Katarakt. Keine wirkliche Verbesserung aufgrund einer sekundären Membranpersistenz
1925	Rezidiv Iritis rechts, sekundäre Membranpersistenz links. Konjunktivitis, Episkleritis	1925 und 1926 4 Kapsulotomien. Glaskörperverlust und Blutungen bei den letzten beiden. Aspirin und Pilokarpin
1928	Episkleritis, Konjunktivitis, Blepharitis	Atropin. Arsen- und Phosphorinjektionen
1930	Erneut Katarakt links. Beide Sehnerven, Makula und periphere Netzhäute waren normal	Horizontale Entfernung der Katarakt links
1931	Episkleritis links mit Sehverschlechterung	–
1932	Totale Katarakt rechts mit sekundärem Glaukom, teilweiser Atrophie der Netzhaut und des Sehnervs	–
1933	Katarakt rechts verkalkt, die Netzhaut war unsichtbar und teilweise atrophisch; es bestand nur noch Lichtwahrnehmung	–

## Andere Erkrankungen

Die mögliche Ursache des chronisch rezidivierenden Augenleidens erschließt sich aus anderen Erkrankungen, die in der Biografie von Joyce dokumentiert sind (Tab. 2). Vor allem die wiederholten venerischen Infektionen führten zu der Annahme einer Syphilis oder eines Reiter-Syndroms [12, 15, 19]. Im Jahr 1904 hatte er Harnröhrenausfluss aufgrund einer als „Gonorrhoe“ bezeichneten venerischen Infektion [15]. Nach seiner Rückkehr nach Triest im Frühsommer 1907 lässt sich vermuten, dass er erneut eine Urethritis hatte, offen oder unerkannt [15]. Nach erneutem Kontakt mit Prostituierten bekam Joyce 1909 ein Rezidiv der Geschlechtskrankheit (vorübergehenden Harnröhrenausfluss) gleichzeitig mit einem Rezidiv der Iritis und einer Ischialgie [15].

**Tab. 2:** Andere Erkrankungen. (Zusammengestellt aus [11, 15, 19, 22, 26])

Jahr	Manifestation	Therapie
1904	Venerische Infektion mit urethritischen Symptomen	–
1907	Erneute venerische Infektion	–
1907	Akute Gelenkerkrankung („rheumatisches Fieber“) „Lähmung“ rechter Arm	Ambulante Behandlung mit Elektrotherapie
1909	Erneut Urethritis. Ischialgie	–
1916	Erneut rheumatische Beschwerden	–
1922	„Spinale Arthritis“	Endokrine Therapie und Zahnpflege

Von besonderer Bedeutung für die diagnostische Einordnung ist der zeitliche Zusammenhang der ersten Manifestation einer Iritis mit einer akuten Gelenkerkrankung 1907, die zu damaliger Zeit als „rheumatisches Fieber“ diagnostiziert wurde. Die ersten rheumatischen Schmerzen traten 5 Tage nach Beginn der Iritis auf [22]. Er war 3 Wochen bettlägerig. Danach hatte er noch Schwierigkeiten beim Gehen und ging gebückt, wie sein Bruder mitteilt [22]. Während der Bettlägerigkeit entstand eine vollständige oder teilweise Lähmung des rechten Armes, die mit einer Elektrotherapie ambulant behandelt wurde [22]. Die in anderen Quellen angegebene stationäre Behandlung [26] war nach Recherche von Schneider in Wirklichkeit eine ambulante Behandlung [22].

Im folgenden Jahr berichtet Joyce über eine Besserung seines „Rheumatismus“, und 1909 war er ohne „rheumatische Beschwerden“. Als neues Symptom manifestierte sich jedoch im gleichen Jahr zusammen mit einer erneut vermuteten Urethritis und einem Rezidiv der Iritis eine Ischialgie entlang des Beines [11]. Im Jahr 1916 kam es wieder zu rheumatischen Beschwerden [11]. Die Diagnose einer „spinalen Arthritis“ wird 1922 mitgeteilt, wobei unklar bleibt, was genau damit gemeint ist [11]. Der amerikanische Endokrinologe Dr. Louis Berman, der bei einem Besuch in Paris Joyce untersuchte, empfahl eine „endokrine“ Therapie und zahnärztliche Versorgung [11]. Worin diese „endokrine“ Therapie bestand und ob sie durchgeführt wurde, ist nicht bekannt.

## Argumente für eine reaktive Arthritis

Wegen seines Kontaktes mit Prostituierten und seiner venerischen Infektionen wurde mehrfach argumentiert, dass Joyce an einer Syphilis erkrankt sei [2, 5, 22]. Deshalb muss diskutiert werden, welche Argumente für oder gegen diese Diagnose sprechen. Dabei wird die Literatur zur Uveitis anterior mit herangezogen, da sie entweder aus einer Iritis oder einer Iridozyklitis besteht und das gleiche ätiologische Spektrum umfasst [10].

Die okuläre Syphilis kann in jedem Stadium (primär, sekundär, latent, tertiär) auftreten und zu Uveitis, Optikusneuropathie, retinaler Vaskulitis, interstitieller Keratitis, Episkleritis oder Skleritis und syphilitischer Konjunktivitis führen [16]. Vor 1940 war Syphilis nach Tuberkulose die zweithäufigste Ursache für eine Uveitis [3]. Die syphilitische Uveitis tritt am häufigsten im sekundären, späten latenten und tertiären Stadium auf [16]. Im Primärstadium sind Augenbeteiligungen selten und manifestieren sich hauptsächlich als Schanker an den Augenlidern und der Bindehaut [7]. Nur in Einzelfällen wird eine Uveitis als initiale und einzige Manifestation beschrieben [29]. Die luetische Iritis dauert in der Regel 2 bis 8 Wochen und rezidiviert nicht im Gegensatz zur „rheumatischen“ Iritis [17]. Im Juni 1930 waren beide Sehnerven, Makula und periphere Netzhäute noch normal [24], was zusammen mit den mehrfachen Rezidiven eindeutig gegen eine okuläre Syphilis spricht. Auch gibt es keine biografischen Mitteilungen, dass Joyce im Rahmen seiner beiden urogenitalen Infektionen Manifestationen einer primären Syphilis am Genitale aufwies. Schließlich fehlen in seiner Krankengeschichte sichere Hinweise auf Symptome einer sekundären oder tertiären Syphilis. Die flüchtige Parese des rechten Armes wurde als wichtiger Nachweis für die Diagnose einer Syphilis gewertet [2, 5, 22]. Eine selbst limitierende motorische Neuropathie kann jedoch ebenso als Manifestation einer ReA auftreten [25].

Insgesamt sprechen die bekannten Fakten gegen eine Syphilis und machen als Ursache eine Erkrankung aus dem Formenkreis der SpA wahrscheinlich, wie in medizinischen Publikationen diskutiert [12, 15, 19, 26]. Die akute anteriore Uveitis ist die häufigste extraartikuläre Manifestation einer SpA mit einer Rezidivhäufigkeit von mindestens 50,6 % und findet sich bei ReA in 25,6 % [31]. Beim Reiter-Syndrom, das wir heute als ReA klassifizieren, wurde eine große Neigung zu multiplen Rezidiven und eine schlechte Prognose hinsichtlich der endgültigen Sehschärfe beschrieben, wie auch der Krankheitsverlauf der Iritis bei Joyce eindrücklich zeigt [23]. Weitere Argumente für eine ReA sind das gleichzeitige Auftreten der Iritis zusammen mit einer Arthritis sowie die Ischialgie und „spinale Arthritis“ als Hinweis auf zeitweise Beteiligung der Wirbelsäule am Krankheitsgeschehen [12]. Dies könnte sogar zu einer typischen Deformierung der Wirbelsäule im Sinne einer Spondylitis ankylosans geführt haben, worauf die Mitteilung hinweist, dass die Haltung von Joyce einem „Fragezeichen“ ähnelte und er in einer Karikatur in stark gebeugter Haltung dargestellt ist [19].

Für die Augenerkrankung bestand eine familiäre Disposition, da der Vater John Stanislaus Joyce wegen Konjunktivitis und Iritis stationär behandelt wurde [11]. Joyce Sohn Giorgio erkrankte mit 30 Jahren an einer als „Rheumatismus“ diagnostizierten Gelenkmanifestation, ein weiterer möglicher Hinweis auf eine familiäre Disposition für eine SpA, die bisher für die Bewertung der Erkrankung von Joyce nicht berücksichtigt wurde [13]. Der genetische Zusammenhang ließe sich jedoch nur durch Human Leukocyte Antigen-B 27-Bestimmungen der Erkrankten und ihrer Nachfahren klären.

Nosologisch ist die Erkrankung von Joyce heute nicht mehr als Reiter-Syndrom zu klassifizieren, da die Bezeichnung wegen der Nazi-Vergangenheit von Hans Reiter abgelehnt wird [9, 27]. Zutreffender ist die diagnostische Zuordnung als chronisch rezidivierende Iritis im Rahmen einer ReA [32]. Auch eine Klassifikation als posturethritische periphere SpA ließe sich rheumatologisch vertreten, da durch die 3 Kriterien Arthritis, Uveitis und vorangegangene urethritische Infektion die Assessment of SpondyloArthritis International Society(ASAS)-Klassifikationskriterien erfüllt werden, auch wenn im Verlauf die Augenerkrankung dominierte [21]. Nach heutigem Kenntnisstand kann als wahrscheinlichster Auslöser der ReA und Iritis eine Infektion mit *Chlamydia trachomatis* angenommen werden, die zu Zeiten von Joyce noch nicht bekannt war, aber mittlerweile eindeutig nachgewiesen ist als Ursache der rheumatischen und okulären Folgeerkrankungen der venerischen Infektion, mitunter auch infolge einer Koinfektion bei Gonorrhö [1, 10, 23, 32].

Schließlich bleibt noch zu diskutieren, warum die Iritis bei Joyce einen so chronischen und ungünstigen Verlauf nahm. Joyce war Raucher, wie ein Porträt belegt (Infobox; [6]). Für die axiale SpA ist Rauchen als ein prognostisch ungünstiger Faktor bekannt [28]. Rauchen wird ebenfalls für die nichtinfektiöse Uveitis als Risikofaktor für Entstehung, Uveitisaktivität, Katarakt, Makulaödem sowie für Rezidive (statistisch nicht signifikanter Trend) beschrieben [4, 20, 30].

### Infobox

James Joyce by Jacques-Emile Blanche, oil on convas 1935. Image ID 3883 © National Portrait Gallery, London

https://www.npg.org.uk/collections/search/portrait/mw03533/James-Joyce

Insgesamt favorisieren die von Medizinern publizierten Analysen der Erkrankung von Joyce ein Reiter-Syndrom [12, 15, 19, 26]. Demgegenüber bevorzugen Kunsthistoriker die Syphilis als Krankheitsursache. Ferris führt als Argumente an: „Joyce litt unter anderem an Magenkrisen als Hinweis auf Tabes dorsalis, das perforiertes Ulkus sei ebenfalls ein Hinweis auf eine Tabes dorsalis, Joyce wurde 1904 Quecksilber verabreicht und später eine dreiwöchige Arseninjektionen, damals die Standardbehandlungen gegen Syphilis.“ [5] Birmingham interpretiert eine medikamentöse Therapie mit Galyl® – ein Arsen-Phosphor-haltiges Produkt – als Beweis für eine Syphilis, während Lyons die Indikation für diese Therapie als tonisierende Medikation beschreibt [2, 14]. Schneider spricht sich in seinen differenzialdiagnostischen Ausführungen dafür aus, dass Joyce 1907 höchstwahrscheinlich an Neurosyphilis erkrankte, wie er aus der Symptomkombination Augen, Parese, rheumatische Symptome, Fieber, Schwierigkeiten oder Unfähigkeit zu gehen, Magenkrämpfe und Neuralgie ableitet [22].

Zusammenfassend fehlen sichere Hinweise auf eine Syphilis, und v. a. die mehrfachen Rezidive der Iritis sind nicht vereinbar mit einer luetischen Infektion. Kunsthistoriker haben auch nach den medizinischen Publikationen, in denen die rheumatische Genese diskutiert wurde, und in deren Kenntnis an der Diagnose der Syphilis festgehalten. Deshalb ist zu wünschen, dass zukünftig die kunsthistorische Literatur die Krankheit von Joyce entsprechend der medizinischen Erkenntnisse wiedergibt. Das zeitliche Zusammentreffen von venerischer Infektion, Arthritis und typischen ärztlich belegten Merkmalen der Iritis sind zusammen mit dem chronisch rezidivierenden Verlauf der Augenerkrankung überzeugende Argumente für eine posturethritische ReA. Diese historische Diagnose bleibt allerdings nur eine Annahme, basierend auf dem heutigen medizinischen Kenntnistand und biografischen Quellen, da detaillierte klinisch rheumatologische Aufzeichnungen und weiterführende Befunde nicht verfügbar sind. Für den die Krankheit prägenden sehr ungünstigen Verlauf der Iritis sind möglicherweise eine familiäre Disposition und das Rauchen verantwortlich.

## Data Availability

Die in dieser Studie erhobenen Datensätze können auf begründete Anfrage beim Korrespondenzautor angefordert werden.
